# LEARNED HELPLESSNESS AMONG ABUSED FILIPINO OLDER PERSONS

**DOI:** 10.1093/geroni/igae098.0956

**Published:** 2024-12-31

**Authors:** Ronaldo Motilla, Carmelina Oyales

**Affiliations:** Philippine Positive Psychology Association, Antipolo City, Rizal, Philippines; Miriam College and Eunoia Educational Consulting, Quezon City, National Capital Region, Philippines

## Abstract

Due to poverty, lack of family and government support, including traumatic abuse experiences and stressful situations, many of the abused Filipino older persons exhibited learned helplessness, which is a belief that they are unable to control or alter their situation even when opportunities for change are available. The feelings of helplessness and hopelessness results in stress, anxiety, depression, isolation, loneliness, lethargy, and even self-harm behaviors and suicidal ideation. The abuses that the older persons experience include but not limited to: physical, psychological/mental, verbal, sexual, neglect, abandonment, financial exploitation and fraud. With the use of qualitative research design, 10 elderly Filipino participants aged 60 and above were recruited through purposive sampling method. Data were collected through the use of in-depth interviews and focused group discussions (FGDs), and case reports. Results were analyze using thematic analysis. Five themes emerged, namely, resignation to fate, fate as an excuse for misfortune, loss of sense of self that is from internal/external conflicts and struggles, stigma and shame. Elder abuse victims tend to accept their circumstances and the situations they are in due to negative mindset, feelings of hopelessness that evolved to their learned helplessness. Keywords: Elder abuse, learned helplessness, aging population, ageism, stigma

